# Copy number variation in Y chromosome multicopy genes is linked to a paternal parent-of-origin effect on CNS autoimmune disease in female offspring

**DOI:** 10.1186/s13059-015-0591-7

**Published:** 2015-02-10

**Authors:** Laure K Case, Emma H Wall, Erin E Osmanski, Julie A Dragon, Naresha Saligrama, James F Zachary, Bernardo Lemos, Elizabeth P Blankenhorn, Cory Teuscher

**Affiliations:** Department of Medicine, University of Vermont, Given Medical Building C317, Burlington, VT 05405 USA; Department of Microbiology and Molecular Genetics, University of Vermont, Burlington, VT 05405 USA; Department of Pathobiology, University of Illinois at Urbana-Champaign, Urbana, IL 61802 USA; Department of Environmental Health, Harvard School of Public Health, Boston, MA 02115 USA; Department of Microbiology and Immunology, Drexel University College of Medicine, Philadelphia, PA 19129 USA; Department of Pathology, University of Vermont, Burlington, VT 05405 USA; Current address: Department of Microbiology and Immunology, Stanford University School of Medicine, Stanford, CA 94305 USA

## Abstract

**Background:**

The prevalence of some autoimmune diseases is greater in females compared with males, although disease severity is often greater in males. The reason for this sexual dimorphism is unknown, but it may reflect negative selection of Y chromosome-bearing sperm during spermatogenesis or male fetuses early in the course of conception/pregnancy. Previously, we showed that the sexual dimorphism in experimental autoimmune encephalomyelitis (EAE) is associated with copy number variation (CNV) in Y chromosome multicopy genes. Here, we test the hypothesis that CNV in Y chromosome multicopy genes influences the paternal parent-of-origin effect on EAE susceptibility in female mice.

**Results:**

We show that C57BL/6 J consomic strains of mice possessing an identical X chromosome and CNV in Y chromosome multicopy genes exhibit sperm head abnormalities and female-biased sex ratio. This is consistent with X-Y intragenomic conflict arising from an imbalance in CNV between homologous X:Y chromosome multicopy genes. These males also display paternal transmission of EAE to female offspring and differential loading of microRNAs within the sperm nucleus. Furthermore, in humans, families of probands with multiple sclerosis similarly exhibit a female-biased sex ratio, whereas families of probands affected with non-sexually dimorphic autoimmune diseases exhibit unbiased sex ratios.

**Conclusions:**

These findings provide evidence for a mechanism at the level of the male gamete that contributes to the sexual dimorphism in EAE and paternal parent-of-origin effects in female mice, raising the possibility that a similar mechanism may contribute to the sexual dimorphism in multiple sclerosis.

**Electronic supplementary material:**

The online version of this article (doi:10.1186/s13059-015-0591-7) contains supplementary material, which is available to authorized users.

## Background

For most autoimmune diseases (AIDs) there is a clear sexual dimorphism (SD) in prevalence, in that females are more frequently affected than males, despite the fact that disease severity is often greater in males [1]. The female preponderance in AIDs has been ascribed to genetic, hormonal, and developmental influences, and target organ susceptibility. However, aside from an ‘immunocompetence handicap’ model [2], which posits that testosterone in males drives the development of secondary male sex characteristics at the expense of suppressing immunity [3], the mechanisms underlying the decreased male prevalence of AIDs are ill defined. Moorthy *et al.* [4] hypothesized that the decreased prevalence of systemic lupus erythematosus (SLE) in men may be due to negative selection of male fetuses possessing genetic risk factors for SLE early in the course of conception and pregnancy. Support for this hypothesis was found in a chart review of patients with childhood onset SLE, systemic onset juvenile rheumatoid arthiritis (SoJRA), and pauciarticular onset juvenile rheumatoid arthritis (PaJRA). SLE and PaJRA exhibit female-biased SD in disease prevalence, whereas SoJRA is a non-SD AID. Their results revealed a significant reduction in the sex ratio for children of SLE and PaJRA families compared with families of patients with SoJRA. Consistent with this observation, Aggarwal *et al*. [5] subsequently reported, using pregnancy histories of both SLE affected and unaffected individuals within SLE families, that there was an excess of male fetal loss in SLE families compared with controls. These findings suggest that genetic risk factors for AIDs may play a role in reproduction, a concept that is consistent with the Red Queen Hypothesis model of sexual reproduction and sex chromosome co-adaptation [6]. More generally, the female-biased SD in prevalence observed in many AIDs may not only reflect negative selection of male fetuses early in the course of conception/pregnancy, but also negative selection on Y-bearing sperm during spermatogenesis.

One outcome of intragenomic conflict is the preferential transmission of genetic elements to offspring at a higher frequency than the expected 1:1 Mendelian ratio [7]. These genetic elements are referred to as transmission distorters and tend to evolve in low or non-recombining regions in the genome [8]. In mice, disequilibrium between X and Y chromosome (ChrX, ChrY) multicopy gene homologues contributes to X-Y intragenomic conflict. This results in sex ratio distortion in favor of the sex chromosome harboring more copies of the distorting gene [9-11]. In addition, X-Y intragenomic conflict leads to sperm head defects [12-15], as well as altered chromatin remodeling and sex chromosome gene expression in developing spermatids [16-19]. Thus, copy number variation (CNV) in ChrY multicopy genes that influence experimental allergic encephalomyelitis (EAE) susceptibility in male mice [20] may also contribute to the paternal parent-of-origin (POO) effect on EAE susceptibility in females [21].

In the autoimmune model of multiple sclerosis (MS), EAE, we identified a paternally transmitted POO effect on EAE susceptibility in female offspring [21]. Among F_2_ intercross progeny generated from EAE-susceptible SJL/J (S) and EAE-resistant B10.S/SgMcdJ (B) mice, central nervous system (CNS) infiltration and damage were only found to be different in female mice from the BS × BS intercross, whose grandsires and sires possessed the SJL/J ChrY [21]. In this study, using C57BL/6 J ChrY consomic strains of mice with CNV in multicopy ChrY genes, including *Sly* and *Ssty1*, both of which are mediators of X-Y intragenomic conflict and influence susceptibility to AIDs in males, we identified X-Y intragenomic conflict influencing the paternal POO effect on EAE susceptibility in female offspring. Moreover, we expanded on the observations of Moorthy *et al.* [4] to include studies on the sex ratios of families with probands affected by MS, rheumatoid arthritis (RA), and type 1 diabetes (T1D). We show that families of probands with AIDs displaying a female-biased SD for prevalence, including MS, SLE, RA, and PaJRA, exhibit a female-biased sex ratio, whereas families of probands affected with non-SD AIDs, including SoJRA and T1D, exhibit an unbiased sex ratio. Taken together, our findings raise the possibility that X-Y intragenomic conflict may contribute to the SD seen in human AIDs.

## Results and discussion

### Differences in EAE susceptibility among female offspring from B6-ChrY consomic strains

To aid in the identification of the genetic mechanism driving the paternal POO effect on EAE in female offspring, we explored the effect of natural genetic variation in ChrY on EAE susceptibility using a panel of ChrY consomic mouse strains purchased from The Jackson Laboratory. These strains include B6-ChrY^129S1^, B6-ChrY^A/J^, B6-ChrY^AKR^, B6-ChrY^BUB^, B6-ChrY^LEWES^, B6-ChrY^MA^, B6-ChrY^MET^, B6-ChrY^PWD^, B6-ChrY^RF^, B6-ChrY^ST^, B6-ChrY^SJL^, B6-ChrY^SWR^, and B6-ChrY^WSB^, where the mouse strain donating ChrY to B6 is indicated in superscript. These consomic strains were generated by breeding male mice possessing either a *Mus musculus domesticus* (AKR, BUB, LEWES, MA, MET, RF, ST, SJL, SWR, and WSB) or *Mus musculus musculus* (129S1, A/J, and PWD) ChrY to B6 female mice [22-24]. Then, through a series of backcrosses of 10 generations or more to B6 females, the genome of the male mice, including the autosomes, ChrX, the peudoautosomal regions of ChrX and ChrY, and the mitochondrial genome, are expected to be >99.9% identical to the B6 genome. This was confirmed by performing a genome-wide single nucleotide polymorphism (SNP) analysis, which did not detect donor strain contamination among any of the ChrY consomic strains (Additional file 1). Therefore, all genetic variation among the males from ChrY consomic strains presumably comes from the non-recombining region of ChrY called the non-pseudoautosomal or male-specific region [25]. Since female mice from B6-ChrY consomic strains do not inherit ChrY from their fathers, female mice arising from these ChrY consomic strains are genetically identical to B6 except they were sired and reared by B6 male mice with ChrY from another mouse subspecies.

**Figure 1:**
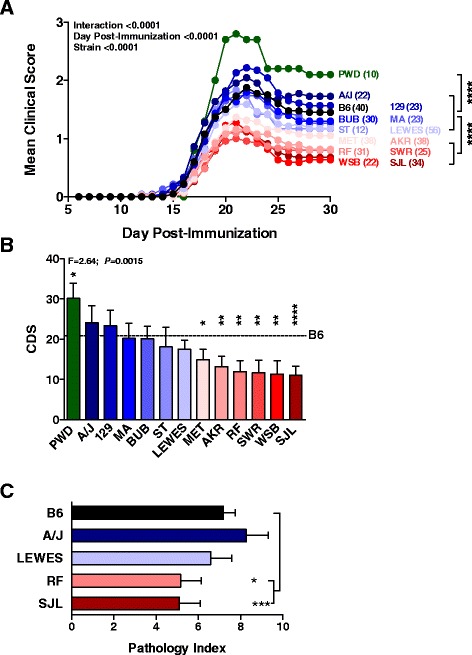
**Polymorphic variation in ChrY influences susceptibility to EAE in female offspring. (A)** Female offspring from B6-ChrY consomic strains were immunized with MOG_35-55_ using the 2× protocol and the clinical score was monitored over 30 days. The consomic strain represented by each line is color coded with the bar graph in (B). Wild-type B6 females are represented by black circles. The significance of the differences in disease course among the strains was determined by two-way ANOVA (interaction (F = 1.517; DFn = 406; DFd = 10,940; *P* < 0.0001), day post-injection (F = 176.1; DFn = 29; DFd = 10,940; *P* < 0.0001), and strain (F = 33.27; DFn = 14; DFd = 10,940; *P* < 0.0001)), followed by Holm-Sidak’s multiple comparisons against B6 (****P ≤ 0.0001). **(B)** The CDS was determined for each strain and the significance of the observed differences determined by one-way ANOVA followed by Holm-Sidak’s multiple comparisons against B6 (*P ≤ 0.05, **P ≤ 0.01; ****P ≤ 0.0001). The x-axis indicates the strain origin of ChrY. The dashed line indicates the CDS for B6. Significance of differences determined by one-way ANOVA followed by Fisher’s LSD test. Data represented as mean ± standard error of the mean. For (A,B), a heterogeneity test was used between cohorts of mice with no significant differences detected. Thus, data were pooled from three independent experiments and the total number of animals analyzed are included in (A). **(C)** Histopathology of the spinal cord from female offspring from B6-ChrY consomic strains immunized using the 2× protocol. Significance of observed differences was determined by one-way ANOVA followed by Holm-Sidak’s multiple comparisons test. n ≥ 15; *P ≤ 0.05, **P ≤ 0.01. Data represented as mean ± standard error of the mean.

**Figure 2:**
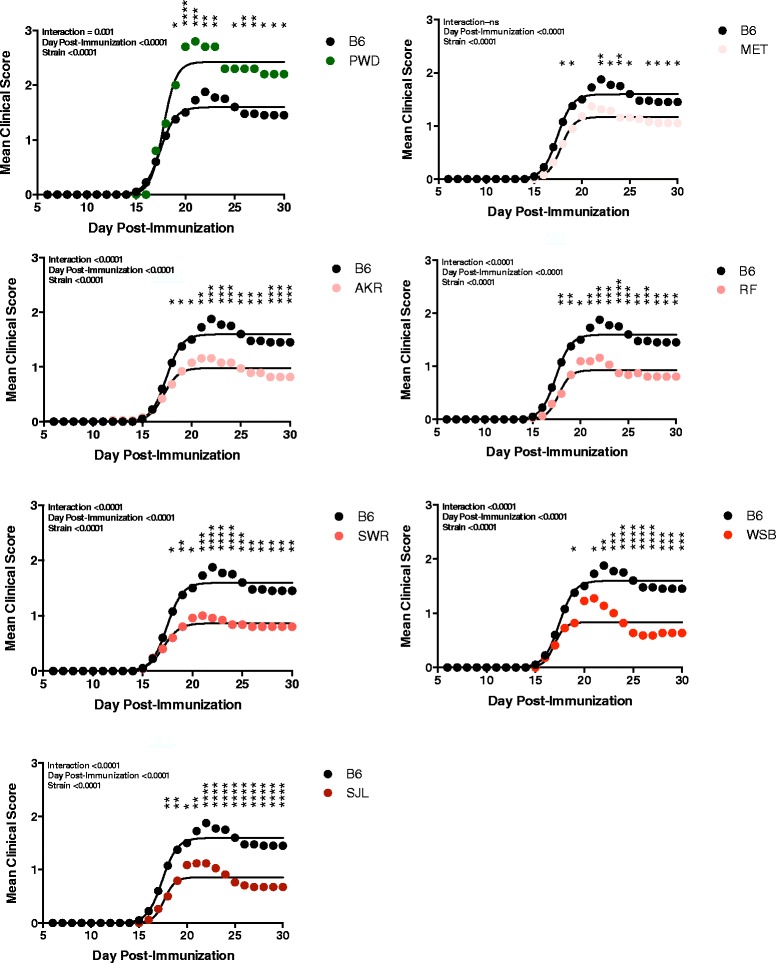
**B6-ChrY consomic strains exhibiting significant differences in EAE severity compared with B6 mice.** Nonlinear regression analysis using the least squares method revealed that, for each strain studied, the disease course elicited fit a sigmoid curve. The significance of the differences in disease course between B6 and each B6-ChrY consomic line was determined by comparing the best-fit values for each line against those of B6 and by two-way ANOVA with an overall adjusted significance threshold of *P* ≤ 0.0001 for effect of strain followed by Holm-Sidak corrected *post hoc* multiple comparisons (*P ≤ 0.05; **P ≤ 0.01; ***P ≤ 0.001; ****P ≤ 0.0001).

EAE was elicited in female mice by immunizing the animals with 100 μg of myelin oligodendrocyte glycoprotein peptide 35-55 (MOG_35-55_) in complete Freund’s adjuvant (CFA) on day 0 and day 7 (2× protocol) and the mice were scored for clinical signs of EAE by recording their disease scores daily for 30 days post-immunization. Analysis of the severity of the clinical disease course (Figure 1A) and the cumulative disease scores (CDSs) (Figure 1B) between female B6 and female mice from each of the B6-ChrY consomic strains was used as measure of disease susceptibility, and revealed a continuous distribution across the strains, consistent with quantitative inheritance. The severity of the clinical disease course for each consomic strain was compared with B6, which revealed that B6-ChrY^A/J^, B6-ChrY^129^, B6-ChrY^MA^, B6-ChrY^BUB^, ChrY^ST^, and B6-ChrY^LEWES^ are equivalently susceptible to EAE as B6 (data not shown). In contrast, B6-ChrY^PWD^ female mice showed an increase in the severity of disease while B6-ChrY^MET^, B6-ChrY^AKR^, B6-ChrY^SWR^, B6-ChrY^WSB^, ChrY^SJL^, and ChrY^RF^ female mice showed a significant reduction in the severity of disease compared with female B6 (Figure 2). Histopathologic analysis of the CNS from two low responder strains, B6-ChrY^SJL^ and B6-ChrY^RF^, revealed significantly less spinal cord neuropathology compared with female B6 (Figure 1C).

**Figure 3:**
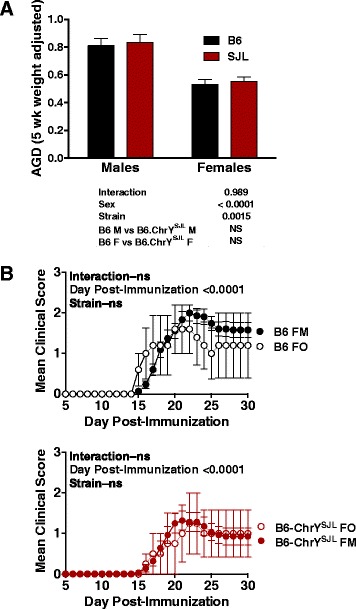
**Evidence that pre- and postnatal exposure to males does not influence EAE susceptibility in female offspring of B6 and B6-ChrY** ^**SJL**^ **strains of mice. (A)** AGD, which is reflective of the level of exposure of females to testosterone during development *in utero*, was determined for female offspring of B6 and B6-ChrY^SJL^. Analysis was carried out using the values for AGD after correcting for the effects of body weight. Significance of observed differences was determined by two-way ANOVA followed by Bonferroni’s multiple comparisons test. n ≥ 55 male or female mice per strain across multiple dams and litters. **(B)** EAE was elicited in adult female mice by immunizing with 100 μg of MOG_35-55_ in CFA on days 0 and 7. Mice were scored for clinical signs of EAE for 30 days. FM, females and males (sire and siblings) co-housed until weaning; FO, female only litters generated by removing the sire and male siblings on day of birth. Significance in disease course was determined by two-way ANOVA. n = 5 mice per strain and data are representative of two independent experiments. Data represented as mean ± standard error of the mean. NS, not significant.

Important variables that need to be considered when analyzing paternal POO effects in female offspring are potential differences in the prenatal and postnatal environments, which may account for the differences in EAE observed for the female offspring of B6-ChrY consomic strains. One potential mechanism whereby ChrY polymorphism could influence EAE susceptibility is through the organizational masculinizing effects of exposure to excess androgen during development [21]. Examining the anogenital distance (AGD) provides a means for retrospectively assessing the organizational masculinization of female fetuses as a consequence of fetal androgen exposure via an intrauterine positional effect [26]. Therefore, to assess whether or not the POO effects elicited by ChrY^SJL^ on EAE susceptibility in female mice is due to differences in androgen exposure during development, we determined the AGD of male and female B6 and B6-ChrY^SJL^ mice at 5 weeks of age. No significant differences in the weight-adjusted AGD between male and female B6 and B6-ChrY^SJL^ mice were detected (Figure 3A).

To directly test the effect of the postnatal environment, we generated 'female only' litters for B6 and B6-ChrY^SJL^ by removing the sire from the cage once the dam was noticeably pregnant and by removing the male siblings from the cage on the day of birth. EAE severity was then compared between female-only litters and conventionally raised females at 10 to 12 weeks of age. Importantly, we did not detect a significant difference in EAE between female mice reared in the presence or absence of their sire and male siblings for the tested strains (Figure 3B). Therefore, natural genetic variation in ChrY influences EAE in female offspring from B6-ChrY consomic strains independently of postnatal male environmental factors. Taken together, these findings suggest that natural genetic variation in ChrY influences EAE susceptibility in female offspring from B6-ChrY consomic strains independently of androgen-mediated organizational masculinization of female fetuses and postnatal male environmental factors.

### ChrY CNV is linked to EAE severity among the female offspring of B6-ChrY consomic strains

Until recently, the murine ChrY was reported to be 15.9 Mb in length with 17 protein coding genes, and this information has contributed to the consensus that ChrY is primarily composed of 'junk' DNA whose contribution to phenotypic differences among the sexes is limited to sexual development and spermatogenesis [27]. Then, the release of the 2012 mouse primary genome assembly by the Genome Reference Consortium (GRCm38) markedly changed the landscape of ChrY. The Ensembl Genome Browser [28], which uses the current assembly, indicates that the mouse ChrY is approximately 91 Mb in length and contains 377 protein coding genes, most of which are classified as predicted protein coding genes and are likely members of multicopy gene families residing in large interrelated repeat element arrays [27]. Finally, recent sequencing of the mouse ChrY suggests there are approximately 700 protein coding genes on the male-specific region of ChrY [29]. This investigation currently provides the most accurate information on the number of ChrY multicopy gene families with homologs on ChrX, including *Sly*, *Rbmy*, *Ssty1*, *Ssty2*, *Rbm31y*, and *Srsy*, which are amplified to varying degrees among different *Mus* species [29].

**Figure 4:**
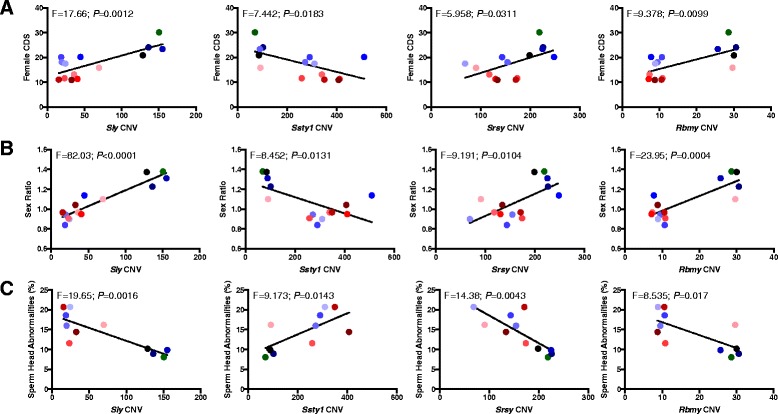
**CNV in ChrY multicopy genes is linked to EAE, sex ratio, and sperm head abnormalities among B6-ChrY consomic strains.** Linear regression analyses of CNV with **(A)** female CDS, **(B)** sex ratio, and **(C)** sperm head abnormalities reveals a significant relationship between the regressed variables. B6-ChrY consomic strains are represented by each data point and are color-coded with the bar graph in Figure 1B.

To investigate the extent of CNV among these multicopy ChrY genes from the B6-ChrY consomic panel of mice, we performed quantitative PCR analysis using the number of gene copies reported for B6 as the reference genome to calculate the number of ChrY gene copies for each of the B6-ChrY consomic strains (Table 1). Among male B6-ChrY consomic strains of mice, linear regression analysis identified the presence of a significant relationship between EAE CDS and CNV in *Sly*, *Ssty1*, *Ssty2*, and *Rbmy* (Table 2). Similarly, among female offspring of B6-ChrY consomic strains of mice, linear regression analysis identified the presence of a significant relationship between EAE CDS and CNV in *Sly*, *Ssty1*, *Srsy*, and *Rbmy* (Table 2 and Figure 4A).

**Table 1:** **Assessment of ChrY gene copy number among B6-ChrY consomic strains**

	***Slx*** ^***b***^	***Sly***	***Ssty1***	***Ssty2***	***Srsy***	***Rbmy***	***Rbm31y***
C57BL/6 J BACs^a^		126	85	221	197	30	2
C57BL/6 J	26 ± 1	128 ± 6	85 ± 4	238 ± 49	199 ± 13	30 ± 1	2 ± 0
B6-ChrY^PWD^	29 ± 2	150 ± 6	70 ± 4	375 ± 33	219 ± 17	29 ± 1	1 ± 0
B6-ChrY^A/J^	26 ± 1	136 ± 9	102 ± 4	287 ± 91	226 ± 7	31 ± 1	2 ± 0
B6-ChrY^129^	29 ± 2	155 ± 9	90 ± 5	620 ± 87	225 ± 21	26 ± 1	2 ± 0
B6-ChrY^MA^	26 ± 1	45 ± 2	510 ± 8	2381 ± 249	248 ± 18	8 ± 0	1 ± 0
B6-ChrY^BUB^	27 ± 2	19 ± 1	290 ± 25	1041 ± 158	143 ± 15	11 ± 0	1 ± 0
B6-ChrY^ST^	24 ± 1	20 ± 1	272 ± 10	366 ± 49	154 ± 5	10 ± 1	1 ± 0
B6-ChrY^LEWES^	26 ± 1	24 ± 1	310 ± 39	87 ± 7	69 ± 5	9 ± 1	1 ± 0
B6-ChrY^MET^	28 ± 2	70 ± 5	91 ± 3	183 ± 16	91 ± 9	30 ± 1	2 ± 0
B6-ChrY^AKR^	27 ± 1	36 ± 4	340 ± 64	237 ± 9	118 ± 13	7 ± 0	1 ± 0
B6-ChrY^SWR^	26 ± 1	23 ± 1	259 ± 8	580 ± 54	174 ± 10	11 ± 1	1 ± 0
B6-ChrY^WSB^	27 ± 1	41 ± 4	411 ± 59	787 ± 93	130 ± 14	7 ± 0	1 ± 0
B6-ChrY^SJL^	27 ± 1	16 ± 1	350 ± 56	1289 ± 194	171 ± 20	11 ± 0	1 ± 0
B6-ChrY^RF^	28 ± 1	33 ± 1	407 ± 18	1503 ± 101	134 ± 10	9 ± 1	1 ± 0

^a^Copy number of ChrY genes determined by sequencing from C57BL/6 J BAC libraries [29]. This number was assigned to a B6 calibrator sample and used to calculate CNV for the ChrY consomic strains.

^b^Estimated copy number of 25 was assigned to a B6 calibrator sample to calculate CNV in *Slx* among female offspring of B6-ChrY consomic strains [29].

Numbers represent the mean copy number ± standard error of the mean rounded to the nearest integer. n = 3 to 5 mice per strain.

**Table 2:** **Linear regression analyses summarizing the significant relationship of ChrY CNV with EAE susceptibility and phenotypes associated with X-Y intragenomic conflict**

**X**	**Female CDS**	**Male CDS**	**Sex ratio**	**Sperm head abnormalities**
*Sly*	F = 17.66; *P* = 0.0012	F = 13.26; *P* = 0.0034	F = 82.03; *P* < 0.0001	F = 19.65; *P* = 0.0016
*Ssty1*	F = 7.442; *P* = 0.0183	F = 12.31; *P* = 0.0043	F = 8.452; *P* = 0.0131	F = 9.173; *P* = 0.0143
*Ssty2*	F = 0.4947; *P* = 0.4953	F = 6.323; *P* = 0.0272	F = 0.1788; *P* = 0.6798	F = 0.8289; *P* = 0.3863
*Srsy*	F = 5.958; *P* = 0.0311	F = 0.5336; *P* = 0.4791	F = 9.191; *P* = 0.0104	F = 14.38; *P* = 0.0043
*Rbmy*	F = 9.378; *P* = 0.0099	F = 8.751; *P* = 0.012	F = 23.95; *P* = 0.0004	F = 8.535; *P* = 0.017
*Rbm31y*	F = 3.017; *P* = 0.108	F = 2.156; *P* = 0.1677	F = 11.14; *P* = 0.0059	F = 3.024; *P* = 0.116

### CNV among *M. m. domesticus* ChrYs leads to X-Y intragenomic conflict during spermatogenesis

**Figure 5:**
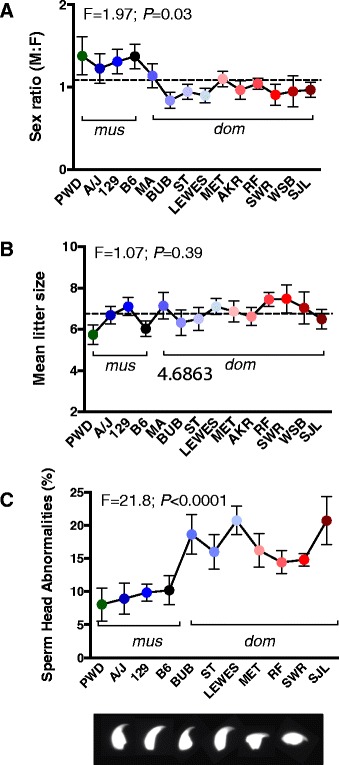
**B6-ChrY consomic strains inheriting a** ***M. mus domesticus*** **ChrY exhibit phenotypes associated with X-Y intragenomic conflict. (A)** Mean sex ratio for each strain was calculated by determining the male:female (M:F) sex ratio across all litters born to a single dam, then averaging the sex ratio across all dams tested. n ≥ 10. Significance of differences determined by one-way ANOVA. **(B)** Mean litter size for each strain was calculated by averaging the number of offspring across all litters born to one dam, then averaging litter size across all dams tested (n ≥ 10). Significance of differences determined by one-way ANOVA. **(C)** The percentage of sperm head abnormalities for each strain. The image depicts a normal sperm head on the left followed by representative pictures of abnormal sperm heads seen across the strains. Significance of differences determined by one-way ANOVA followed by Dunnett’s multiple comparisons test. *P* ≤ 0.05 for all *domesticus* B6-ChrY consomic strains. Data are represented as the mean ± standard deviation. n ≥ 5 mice per strain.

Among the multicopy genes showing a significant association in both male and female CDS, CNV in *Sly* and *Ssty1* are both ascribed mediators of intragenomic conflict during spermatogenesis [9,30]. To evaluate whether the copy number differences were sufficient to elicit X-Y intragenomic conflict during spermatogenesis among the B6-ChrY consomic strains, we evaluated phenotypes associated with disequilibrium between X-Y homologues, including sex ratio distortion and sperm head abnormalities. We observed a significant sex ratio bias in favor of females, but no difference in the mean litter size, among B6-ChrY consomic strains inheriting a *M. m. domesticus* ChrY (Figure 5A,B). Furthermore, we observed an increase in the percentage of sperm head abnormalities among B6-ChrY consomic strains inheriting a *M. m. domesticus* ChrY (Figure 5C) [9]. We performed multiple regression analyses between each of these phenotypes and ChrY multicopy gene number as predictors of X-Y intragenomic conflict and identified a significant relationship between *Sly*, *Ssty1*, *Srsy*, and *Rbmy* CNV with both sex ratio and sperm head abnormalities (Table 2 and Figure 4B,C). These data support a role for X-Y intragenomic conflict during spermatogenesis among B6-ChrY consomic strains inheriting a *M. m. domesticus* ChrY. Furthermore, regression analyses between EAE CDS for either males or females with sex ratio and sperm head abnormalities identified a significant relationship across all regressed variables, thus providing support for X-Y intragenomic conflict as the underlying factor driving the paternal POO effect on EAE among female offspring of B6-ChrY consomic mice (Table 3). However, whether X-Y intragenomic conflict is the result of CNV in *Sly* and *Ssty1*, which are the ascribed mediators of X-Y intragenomic conflict [9,30], or whether other ChrY multicopy genes contributes to this phenomenon remains to be determined. Of note, we have excluded the potential for acquired CNV in *Slx*, the ChrX homolog of *Sly*, among the consomic strains, as they all possess similar numbers of *Slx* copies for up to three generations of brother-sister matings (Table 1).

**Table 3:** **Linear regression analyses summarizing the significant relationship between EAE susceptibility and phenotypes associated with X-Y intragenomic conflict**

	**Female CDS**	**Male CDS**	**Sex ratio**	**Sperm head abnormalities**
Female CDS		F = 9.063; *P* = 0.0109	F = 11.97; *P* = 0.0047	F = 4.951; *P* = 0.05
Male CDS	F = 9.063; *P* = 0.0109		F = 7.130; *P* = 0.0204	F = 10.88; *P* = 0.0093
Sex ratio	F = 11.97; *P* = 0.0047	F = 7.130; *P* = 0.0204		F = 16.14; *P* = 0.003
Sperm head abnormalities	F = 4.951; *P* = 0.05	F = 10.88; *P* = 0.0093	F = 16.14; *P* = 0.003	

### Differential expression of microRNA species within mature sperm

We examined whether ChrY polymorphism may affect other features of mature sperm in addition to sperm morphology, specifically the presence of microRNAs (miRNAs) that are packaged within sperm during spermatogenesis [31,32]. Using miRNA microarrays, we analyzed the miRNA content in sperm from B6 and two B6-ChrY consomic strains in which CNV is low and females exhibit a significant reduction in EAE severity. To this end, triplicate samples of mature sperm from the cauda epididymis and vas deferens following the double swim-out method were pooled from five male B6, B6-ChrY^SJL^, or B6-ChrY^RF^ mice for each replicate and miRNA expression was assessed.

Principal component analysis (PCA) was conducted to describe the variation in the total miRNA data and it was able to clearly recapitulate the sample groups through PC1, which accounted for 29% of the variation. The remaining variation was spread across several components that appear to reflect within-sample group differences, with the B6 sample group exhibiting the largest variation. Of the 1,412 *Mus musculus* miRNAs present on the chip, a binary filter of *P* < 0.05 and fold change ≥2 identified 49 miRNA with differential expression in B6-ChrY^RF^ versus B6 and 23 miRNAs with differential expression in the B6-ChrY^SJL^ versus B6 comparison (Additional file 2). Fifteen of the differentially expressed miRNAs were common between the two comparisons. These data provide evidence that ChrY polymorphism influences differential miRNA processing and/or packaging in the sperm of B6, B6-ChrY^RF^, and B6-ChrY^SJL^ mice. Whether the differences in sperm miRNA content play a role in mediating the paternal POO effect on EAE in female offspring remains to be determined.

### Naïve CD4^+^ T cells from adult B6-ChrY^SJL^ mice exhibit reduced cytokine production upon stimulation

Previously, we identified profound differences in gene expression and alternative splicing in naive CD4^+^ T cells from male B6-ChrY^SJL^ consomic mice compared with B6 [20]. Considering the parallels in EAE susceptibility between male and female B6-ChrY^SJL^ consomic mice, and that CD4^+^ T cells are considered to be central mediators of MS and EAE pathogenesis [33,34], we hypothesized that we may detect changes in the basal transcriptome of CD4^+^ T cells from female offspring of B6-ChrY^SJL^ mice. To address this, RNA was isolated from CD4^+^TCRβ^+^ cells obtained using fluorescently activated cell sorting (FACS) from the lymph nodes of adult (8 weeks of age) naïve female B6-ChrY^SJL^ and B6 mice and mRNA expression and the presence of alternative splice variants were assessed.

The PCA map of the CD4^+^TCRβ^+^ transcriptome recapitulated the sample groups through PC1, which accounted for 24.4% of the variation. The remaining variation was spread across several components that appear to reflect within-sample group differences, with the B6 sample group exhibiting the largest variation. Filtering the gene array data using a conservative false discovery rate of <0.05 failed to identify any differentially expressed transcripts between B6-ChrY^SJL^ and B6 CD4^+^ T cells. However, 362 alternatively spliced transcripts passed a false discovery rate <0.05 (Additional file 3). These alternatively spliced genes are distributed throughout the genome and reside on all autosomes and ChrX.

**Figure 6:**
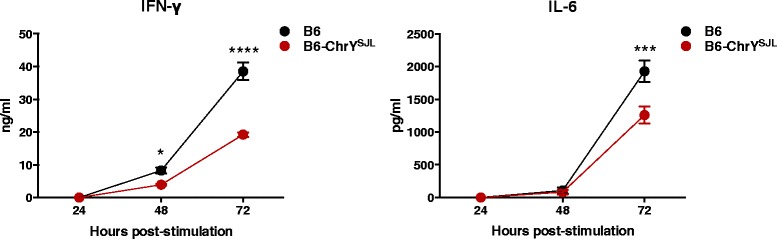
**Natural polymorphic variation in ChrY influences cytokine profiles of activated CD4** ^**+**^ **T cells from female offspring of B6-ChrY** ^**SJL**^ **consomic mice.** Purified CD4^+^ T cells from B6-ChrY^SJL^ and B6 lymph nodes were incubated with anti-CD3 and anti-CD28 monoclonal antibodies and the supernatants were tested for IFN-γ, IL-6, and IL-17 production after 24, 48, and 72 hours by ELISA. Data are representative of three independent experiments. IL-17 was below the limit of detection. Significance determined by two-way ANOVA followed by Holm-Sidak’s multiple comparisons test. *P ≤ 0.05; ***P ≤ 0.001; ****P ≤ 0.0001.

To determine whether there were any intrinsic changes in T-cell signaling between CD4^+^ T cells from female offspring of B6-ChrY^SJL^ versus B6 mice, we purified CD4^+^ T cells from the lymph nodes, stimulated them with anti-CD3 and anti-CD28 monoclonal antibodies, and tested the supernatants for IL-17, IFN-γ, and IL-6 production after 24, 48, and 72 hours by enzyme-linked immunosorbent assay (ELISA). We observed significant reductions in IFN-γ and IL-6 cytokine secretion by stimulated CD4^+^ T cells from B6-ChrY^SJL^ compared with B6 (Figure 6), and IL-17 was consistently below the limits of detection. These data indicate that the ChrY-mediated paternal POO effect also influences biological changes in T-cell responses.

### Female sex-ratio bias among families of probands affected by SD AIDs

Many AIDs are sexually dimorphic with a female bias predominating, but the degree of the sex bias varies greatly depending on the AID [1]. Systemic AIDs, such as SLE and Sjogren’s syndrome, exhibit a strong female sex bias of 9:1. Other AIDs exhibit a more modest female sex bias, like MS and RA, while some diseases exhibit no sex bias, such as T1D and Crohn’s disease. To expand on the findings of Moorty *et al*., we calculated the sibling sex ratios of families with probands for MS, RA, and T1D. We found sex ratio distortion in favor of females only in SD AIDs, including MS, RA, SLE, and PaJRA, but not within families of probands affected with non-SD AIDs, including SoJRA and T1D (Table 4). Taken together, our findings suggest that the female-biased SD in prevalence observed in many AIDs may not only reflect negative selection of male fetuses early in the course of conception/pregnancy [5], but also negative selection on Y-bearing sperm during spermatogenesis.

**Table 4:** **Sibling sex ratios of families with probands with SD or non-SD AIDs**

**Autoimmune disease**	**Sexual dimorphism**	**Male**	**Female**	**Sex ratio**
**Multiple sclerosis**	F > M			
Jorge Oksenberg (personal communication)		531	1,356	0.39
Bertrand Fontaine (personal communication)		252	405	0.62
Sawcer *et al*. [35]		190	388	0.49
Ebers *et al*. [36]		220	366	0.6
**Systemic lupus erythematosus**	F > M			
Aggarwal *et al*. [5]		3,201	5,434	0.59
Moorthy *et al*. [4]		115	181	0.64
**Rheumatoid arthritis**	F > M			
Lindsey Criswell (personal communication) [37]		987	1,633	0.6
Peter Gregersen (NARAC)		2,032	3,087	0.66
**Pauciarticular onset juvenile arthritis**	F > M	62	114	0.54
Moorthy *et al*. [4]				
**Systemic onset juvenile arthritis**				
Moorthy *et al*. [4]	F = M	32	32	1
**Type 1 diabetes**	F = M			
Patrick Concannon (personal communication)		1,589	1,489	1.07
John Todd (personal communication)		3,707	3,627	1.07

F, female; M, male.

## Conclusions

Our data show that CNV between sex chromosome multi-copy genes results in X-Y intragenomic conflict during spermatogenesis and is significantly linked to differences in EAE severity among female offspring of B6-ChrY consomic strains. The mammalian sex chromosome interaction is reminiscent of interactions between the ChrX and ChrY encoded multi-copy genes *Stellate* (*Ste*) and *Suppressor of Stellate* (*Su(ste)*) in *Drosophila*. Deletions of the *Drosophila* Y-linked *Su(ste)* locus cause spermatogenenic phenotypes, and it has been suggested that interactions between *Ste* and *Su(ste)* during spermatogenesis might influence the sex ratio through the differential viability of X-bearing and Y-bearing gametes [38,39]. However, the mechanisms through which *Su(ste)* suppresses the *Ste* locus on ChrX remain poorly elucidated. Similarly, the mechanism by which ChrY CNV impacts the paternal POO effect on EAE in female offspring remains unknown.

Whether X-Y intragenomic conflict occurs in humans remains unknown, although our data showing that families of probands with female-biased SD in AID prevalence (MS, SLE, RA, and PaJRA) also exhibit a female-biased sex ratio, whereas families of probands affected with non-SD AIDs (SoJRA and T1D) exhibit unbiased sex ratios, suggests that X-Y intragenomic conflict may play a role in AID susceptibility in humans. Furthermore, as in *Drosophila* and mice, the human ChrY contains over 10 Mb of ampliconic sequence containing multicopy gene families critical for spermatogenesis, some of which are amplified versions of ChrX homologues with testis-specific expression patterns, that represent potential candidates for X-Y intragenomic conflict [40]. Thus, we provide evidence for a genetic mechanism at the level of the gamete that contributes to the paternal POO effect on EAE susceptibility in adult female mice, raising the possibility that a similar mechanism may contribute to the SD in MS susceptibility.

## Materials and methods

### Mice

All mice in this study were bred and maintained in the animal facility at the University of Vermont. The B6-ChrY consomic panel of mice used in this study was purchased from The Jackson Laboratory (Bar Harbor, ME, USA). The number of backcrosses performed prior to cryopreservation is indicated next to each strain when this information was available on The Jackson Laboratory website [41]. Males from each consomic strain were backcrossed to female C57BL/6 J mice for two generations prior to experimentation. C57BL/6 J (B6), C57BL/6 J-ChrY^129S1/SvImJ^/NaJ (B6-ChrY^129^), C57BL/6JEi-ChrY^A/J^/EiJ (B6-ChrY^AJ^) N17, C57BL/6JEi-ChrY^AKR/J^/EiJ (B6-ChrY^AKR^) N73, C57BL/6JEi-ChrY^BUB/BnJ^/EiJ (B6-ChrY^BUB^) N22, C57BL/6JEi-ChrY^LEWES^/EiJ (B6-ChrY^LEWES^) N33, C57BL/6-ChrY^PWD/Ph^/ForeJ (B6-ChrY^PWD^) N14, C57BL/6JEi-ChrY^RF/J^/EiJ (B6-ChrY^RF^) N22, C57BL/6JEi-ChrY^SJL/J^/EiJ (B6-ChrY^SJL^) N20, C57BL/6JEi-ChrY^ST/bJ^/EiJ (B6-ChrY^ST^) N20, C57BL/6JEi-ChrY^WSB/Ei^/EiJ (B6-ChrY^WSB^) N11, B6Ei.MA-*A*ChrY^MA/MyJ^/EiJ (B6-ChrY^MA^) N19, and B6Ei.SWR-*A*ChrY^SWR/J^/EiJ (B6-ChrY^SWR^) N20. All B6-ChrY consomic strains were maintained by breeding siblings for up to three generations and then backcrossing to B6 mice purchased from The Jackson Laboratory. Animals were housed in specific pathogen-free conditions under National Institutes of Health guidelines, and all experiments performed in this study were approved by the Animal Care and Use Committee of the University of Vermont.

### Genome-wide SNP analysis

DNA isolated from the male B6-ChrY consomic mice purchased from The Jackson Laboratory was tested for donor strain contamination using the Mouse Universal Genotyping Array (MUGA) platform (GeneSeek, Inc., Lincoln, NE, USA). MUGA is a 7,851 SNP marker genotyping array built on the Illumina Infinium platform. SNP markers are distributed throughout the mouse genome with an average spacing of 325 kb.

### Induction and evaluation of EAE

Mice were immunized for the induction of EAE using the double-inoculation (2×) protocol. Mice were injected subcutaneously with a sonicated emulsion of 100 μg of MOG_35-55_ in combination with an equal volume of CFA containing 200 μg of *Mycobacterium tuberculosis* H37RA (Difco Laboratories, Detroit, MI, USA) in the posterior right and left flank; one week later all mice were similarly injected at two sites on the right and left flank anterior of the initial injection sites. Mice were scored daily starting at day 12 post-injection and clinical quantitative trait variables, including disease incidence, mean day of onset, cumulative disease score, number of days affected, overall severity index, and peak score, were generated [42].

CNS histopathologic evaluations were done as previously described [43]. Briefly, brains and spinal cords were dissected on the 30th day post-immunization, fixed in 10% phosphate-buffered formalin, and sections were stained with hematoxylin and eosin and representative areas of the brain and spinal cords were scored in a semiquantitative fashion for the various lesions. The following components of the lesions were assessed: 1) severity of the lesion as represented by each component of the histopathological assessment; 2) extent and degree of myelin loss and tissue injury (swollen axon sheaths, swollen axons, and reactive gliosis); 3) severity of the acute inflammatory response (predominantly neutrophils); and 4) severity of the chronic inflammatory response (lymphocytes/macrophages). A score was assigned separately to the entire brain and spinal cord for each lesion characteristic based on a subjective scale ranging from 0 to 5. A score of 0 indicates no lesions; 1 indicates minimal; 2, mild; 3, moderate; 4, marked; and 5, severe lesions.

### Anogenital distance

AGD was measured from the base of the genital area to the base of the anus at 5 weeks of age using a standard millimeter ruler. One experimenter measured AGD throughout the study to reduce error. Analysis was carried out using the AGD values after correcting for body weight. AGD measurements for both males and females were generated using offspring from multiple dams across multiple litters.

### Gene copy number assay

Tail DNA was isolated from a minimum of three male mice from each B6-ChrY consomic line and wild-type B6 for ChrY CNV analysis. Tail DNA was isolated from a minimum of three female offspring of B6-ChrY consomic strains after three generations of brother-sister matings for ChrX *Slx* CNV analysis. DNA was quantified by Nanodrop and diluted to 5 ng/μl in nuclease-free H_2_O. FAM dye-labeled PCR primers for each ChrY multicopy gene were designed using the Custom Taqman® Assay Design Tool on the Life Technologies website by entering the DNA sequence obtained from Mouse Genome Informatics. VIC dye-labeled mouse *Tfrc* Taqman® Copy Number Reference Assay was used in the duplex real-time PCR reaction and run on the 7500 real-time PCR system according to the manufacturer’s instructions. Data were analyzed using ABI Prism® SDS 2.1 software followed by the CopyCaller® Software Version 2.0 (Life Technologies, Carlsbad, CA, USA). The ΔCt mean for the B6 calibrator sample was subtracted from the ΔCt mean for each B6-ChrY consomic sample, generating the ΔΔCt value for the sample. Copy number was then calculated using the following equation: copy number = cn_c_2^-ΔΔCt^, where cn_c_ is the copy number for B6 as depicted in Table 1 [29].

### CD4^+^ T-cell activation and ELISA

Three biological replicates of CD4^+^ T cells were purified from axillary, brachial, and inguinal lymph nodes pooled from five mice using the EasySep mouse CD4^+^ T-cell enrichment kit and EasySep magnet following the manufacturer’s instructions (Stemcell Technologies, Vancouver, Canada). Nonpolarized effector T cells were generated as previously described [44]. Supernatants were removed after 24, 48 and 72 hours and frozen at -80°C until cytokine levels were tested by ELISA.

### Sperm morphology

Epidydimal sperm was collected using the double swim-out method. The large pieces of tissue were removed using forceps, and the remaining media was filtered through 150 μM mesh into a 15 ml tube. Sperm were pelleted by centrifugation and resuspended in 1 ml of methanol/acetic acid (3:1) fixative and incubated on ice for 5 minutes. Sperm were streaked onto glass slides, allowed to air dry, and mounted using Vectashield mounting medium with DAPI (Vector Laboratories, Burlingame, CA, USA). Morphology was assessed using an Olympus BX50 light microscope with a 60× objective under oil and UV light. Images of the sperm were captured and the number of grossly abnormal sperm as described by Riel *et al*. [16] was assessed by averaging the scoring of 300 sperm per male by two separate individuals. The average perventage coefficient of variation between the two individuals was 9.3.

### FACS-purified CD4^+^ T cells

Three biological replicates were pooled from the axillary, brachial, and inguinal lymph nodes from fvie mice for each replicate. Cells were washed with phosphate-buffered saline (PBS)/1% fetal calf serum (FCS), pelleted at 300 g for 5 minutes at 4°C. A working stock of Live/Dead-775 dye (Invitrogen L10119) was made by adding 1 μl of reconstituted dye to 1 ml PBS and cells were resuspended in 100 μl of working stock per 1 × 10^6^ cells. Cells were incubated for 30 minutes on ice in the dark. Cells were washed with cold PBS/1% FCS, pelleted, and resuspended in 100 μl of an antibody mixture containing 1 μg/ml PerCp-Cy5.5 anti-mouse TCRβ chain and 1 μg/ml Alexa Flour® 488 anti-mouse CD4 antibodies in PBS/1% FCS. Cells were incubated on ice in the dark for 30 minutes and then washed with cold PBS/1% FCS, pelleted, and resuspended at a concentration of 10 × 10^6^ cells per 1 ml of PBS/10% FCS. Cells were filtered through 50 μm nylon mesh and sorted using BD FACSAria to collect approximately 3 × 10^6^ CD4^+^ T cells per sample.

### RNA isolation

RNA was isolated using the Qiagen RNeasy® Plus Mini Kit following the manufacturer’s instructions (Qiagen, Valencia, CA, USA). RNA was eluted using 30 μl RNase-free H_2_O and 0.5 μl Superase (Life Technologies) was added to each sample. RNA was quantified on ND 1000 spectrophotometer v.3.3.1 (ThermoScientific, Wilmington, DE, USA) and quality assessed on a 2100 Bioanalyzer (Agilent, Palo Alto, CA, USA). RNA was stored at -80°C and submitted to the Vermont Genetics Network Microarray Facility.

### Mouse gene array target preparation

Oligonucleotide microarray analysis of RNA expression levels was performed in the Vermont Genetics Network Microarray Facility using the Affymetrix GeneChip Platform (Affymetrix Inc., Santa Clara, CA, USA) according to the manufacturer’s protocols. In brief, the Nugen Ovation system v.2 with SPIA® RNA amplification was employed to convert 50 ng of total RNA to cDNA. This isothermal RNA amplification system produces 5 to 12 μg of anti-sense cDNA targets that is followed by several steps to produce sense strand cDNA to be fragmented, biotinylated and hybridized to the genechip. After purification and fragmentation, biotinylated-cDNA targets were hybridized to the Mouse Gene 1.0 ST Arrays oligonucleotide arrays for 16 hours at 45°C. Hybridized arrays were washed and stained with streptavidin-phycoerythrin followed by sequential incubations with biotin-coupled polyclonal anti-streptavidin antibody and streptavidin phycoerythrin as a fluorescent amplification step. After staining, arrays were scanned (3000-7G Scanner, Affymetrix Inc.) and data collected for statistical analysis.

### Mouse miRNA array target preparation

Epidydimal sperm were treated with somatic cell lysis buffer (0.1% SDS, 0.5% Triton X-100) and miRNA was isolated using the mirVana miRNA isolation method for enrichment of small RNAs (Life Technologies). Total RNA containing small RNA was labeled using the Flashtag RNA labeling kit (Genisphere, Hatfield, PA, USA) and performed in the Vermont Genetics Network Microarray Facility as previously described [20].

### Calculation of probe set statistics

Probe-level intensities were calculated using the Robust Multichip Average (RMA) algorithm, including background correction, normalization (quantile), and summarization (median polish), for each probe set and sample, as is implemented in Partek Genomic Suites®, version 6.6 (Partek Inc., St Louis, MO, USA). Sample quality was assessed based on the relative log expression, and normalized unscaled standard error. PCA of the dataset was also used to look for outlier samples that would potentially introduce latent variation into the analysis of differential expression across sample groups. Linear modeling of sample groups and identification of alternatively spliced variants was performed using ANOVA as implemented in Partek Genomic Suites. The magnitude of the response (fold change calculated using the least square mean) and the *P*-value associated with each probe set and binary comparison were calculated, as well as a step-up, adjusted *P*-value for the purpose of controlling the false discovery rate [45].

### Human sex ratio data

Birth sex ratio data were obtained for each human autoimmune disease presented in Table 4 by using the complete family structures for all respective patients and multiplex families. As with studies looking at the birth sex ratios in SLE [4,5], the total number of male and female siblings for probands with each autoimmune disease was calculated and male-to-female sex ratios generated by dividing the number of male siblings by the number of female siblings.

### Statistics

The statistical analyses for EAE clinical disease traits and sex ratio data were performed using SAS version 9.2 and 9.3, respectively (SAS Institute Inc., Cary, NY, USA). All other analyses were performed using GraphPad Prism version 6 (GraphPad Software, San Diego, CA, USA). The specific tests used are detailed in the figure legends. A *P*-value of ≤0.05 was considered significant.

### Data access

The microarray data discussed in this manuscript have been deposited in the NCBI Gene Expression Omnibus (GEO) [46] and are accessible through GEO SuperSeries accession number GSE65048 [47].

## Additional files

The following additional data files are available with the online version of this paper. Additional file 1:
**Table containing Mouse Universal Genotyping Array SNP data for B6-ChrY consomic strains.**
Additional file 2:
**List of the differentially expressed miRNAs for B6-ChrY** ^**SJL**^ **versus B6 and B6-ChrY** ^**RF**^ **versus B6 sperm microarray dataset, including** ***P*** **-values and fold changes.**
Additional file 3:
**List of alternatively spliced transcripts between B6-ChrY** ^**SJL**^ **and B6 CD4** ^**+**^ **T cells.**

## Abbreviations

AGD: anogenital distance
AID: autoimmune disease
B6: C57BL/6 J
B6-ChrY: C57BL/6 J Y chromosome consomic strains
CDS: cumulative disease score
CFA: complete Freund’s adjuvant
ChrX: X chromosome
ChrY: Y chromosome
CNS: central nervous system
CNV: copy number variation
EAE: experimental allergic encephalomyelitis
ELISA: enzyme-linked immunosorbent assay
FACS: fluorescently activated cell sorting
FCS: fetal calf serum
IFN: interferon
IL: interleukin
miRNA: microRNA
MOG_35-55_: myelin oligodendrocyte glycoprotein peptide 35-55
MS: multiple sclerosis
PaJRA: pauciarticular onset juvenile arthritis
PBS: phosphate-buffered saline
PCA: principal component analysis
POO: parent-of-origin
RA: rheumatoid arthritis
SD: sexual dimorphism/sexually dimorphic
SLE: systemic lupus erythematosus
SNP: single nucleotide polymorphism
SoJRA: systemic onset juvenile arthritis
T1D: type 1 diabetes
