# Supplementary material for: Copy number variation in Y chromosome multicopy genes is linked to a paternal parent-of-origin effect on CNS autoimmune disease in female offspring
Source: Genome Biol. 2015 Feb 10;16(1):28. doi: 10.1186/s13059-015-0591-7 (PMC4396973; doi:10.1186/s13059-015-0591-7)
Supplement: Additional file 2: — List of the differentially expressed miRNAs for B6-ChrY SJL versus B6 and B6-ChrY RF versus B6 sperm microarray dataset, including P -values and fold changes. [file 13059_2015_591_MOESM2_ESM.docx]

| Differentially Expressed Sperm miRNAs – B6-ChrY^SJL^ vs. B6 | | | | |
| --- | --- | --- | --- | --- |
| Affymetrix Probeset ID | **miRNA Symbol** | **Fold Change** | **p-value** | **Description** |
| mmu-miR-128_st | N/A | 2.17398 | 0.0363645 | SJL up vs. B6 |
| mmu-miR-140_st | miR-140-5p (and other miRNAs w/seed AGUGGUU) | 2.00772 | 0.0294944 | SJL up vs. B6 |
| mmu-miR-148b_st | miR-148a-3p (and other miRNAs w/seed CAGUGCA) | 2.99195 | 0.0246266 | SJL up vs. B6 |
| mmu-miR-192_st | miR-192-5p (and other miRNAs w/seed UGACCUA) | 2.11009 | 5.24E-05 | SJL up vs. B6 |
| mmu-miR-301a_st | miR-130a-3p (and other miRNAs w/seed AGUGCAA) | 2.56787 | 0.0155232 | SJL up vs. B6 |
| mmu-miR-30e-star_st | miR-30a-3p (and other miRNAs w/seed UUUCAGU) | 2.67058 | 0.0129912 | SJL up vs. B6 |
| mmu-miR-320_st | miR-320b (and other miRNAs w/seed AAAGCUG) | -2.24578 | 2.62E-06 | SJL down vs. B6 |
| mmu-miR-375_st | miR-375-3p (and other miRNAs w/seed UUGUUCG) | 2.57875 | 0.00656423 | SJL up vs. B6 |
| mmu-miR-210_st | miR-210-3p (and other miRNAs w/seed UGUGCGU) | -2.07096 | 0.00161906 | SJL down vs. B6 |
| mmu-miR-466e-3p_st | miR-297a-3p (and other miRNAs w/seed AUACAUA) | 2.3024 | 0.0480705 | SJL up vs. B6 |
| mmu-miR-34b-5p_st | miR-34a-5p (and other miRNAs w/seed GGCAGUG) | 2.06895 | 0.0192813 | SJL up vs. B6 |
| mmu-miR-465a-3p_st | miR-465b-3p (and other miRNAs w/seed AUCAGGG) | 2.08868 | 0.0261463 | SJL up vs. B6 |
| mmu-miR-465a-5p_st | miR-465b-5p (and other miRNAs w/seed AUUUAGA) | 3.87336 | 0.0104721 | SJL up vs. B6 |
| mmu-miR-465b-3p_st | N/A | 2.25858 | 0.0194369 | SJL up vs. B6 |
| mmu-miR-465c-3p_st | N/A | 2.08372 | 0.0225991 | SJL up vs. B6 |
| mmu-miR-465c-5p_st | N/A | 3.87766 | 0.00239994 | SJL up vs. B6 |
| mmu-miR-467c_st | miR-467c-5p (and other miRNAs w/seed AAGUGCG) | 2.22652 | 0.023487 | SJL up vs. B6 |
| mmu-miR-467e_st | miR-467e-5p (and other miRNAs w/seed UAAGUGU) | 2.55823 | 0.0355901 | SJL up vs. B6 |
| mmu-miR-493_st | miR-493-3p (and other miRNAs w/seed GAAGGUC) | -3.60841 | 0.00134193 | SJL down vs. B6 |
| mmu-miR-671-3p_st | miR-671-3p (and other miRNAs w/seed CCGGUUC) | -2.07114 | 0.0143378 | SJL down vs. B6 |
| mmu-miR-741_st | miR-741-3p (and other miRNAs w/seed GAGAGAU) | 2.33176 | 0.0284476 | SJL up vs. B6 |
| mmu-miR-743b-3p_st | miR-743b-3p (and other miRNAs w/seed AAAGACA) | 2.23003 | 0.0242328 | SJL up vs. B6 |
| mmu-miR-883a-3p_st | miR-883-3p (and other miRNAs w/seed AACUGCA) | 2.37675 | 0.0255776 | SJL up vs. B6 |

| Differentially Expressed Sperm miRNAs – B6-ChrY^RF^ vs. B6 | | | | |
| --- | --- | --- | --- | --- |
| Affymetrix Probeset ID | **miRNA Symbol** | **Fold Change** | **p-value** | **Description** |
| \| hp_mmu-mir-133b_st \| 2.02728 \| \| --- \| --- \| | mir-133 | 2.02728 | \| 0.00140587 \| RF up vs B6 \| \| --- \| --- \| | RF up vs. B6 |
| hp_mmu-mir-468_st | Mir468 | \| 2.48475 \| 0.00431036 \| RF up vs B6 \| \| --- \| --- \| --- \| | 0.00431036 | RF up vs. B6 |
| hp_mmu-mir-669f_st | mir-467 | 2.2459 | 0.0147843 | RF up vs. B6 |
| mmu-let-7f-star_st | let-7a-3p (and other miRNAs w/seed UAUACAA) | -2.71612 | 0.0113467 | RF down vs. B6 |
| mmu-miR-1_st | miR-1-3p (and other miRNAs w/seed GGAAUGU) | 2.3466 | 0.0391621 | RF up vs. B6 |
| mmu-miR-10a-star_st | miR-10a-3p (and other miRNAs w/seed AAAUUCG) | -2.0747 | 0.0129035 | RF down vs. B6 |
| mmu-miR-1306_st | miR-1306-3p ( and other miRNAs w/seed CGUUGGC) | -2.30138 | 0.00234976 | RF down vs. B6 |
| mmu-miR-141_st | miR-141-3p (and other miRNAs w/seed AACACUG) | -2.12596 | 0.00981947 | RF down vs. B6 |
| mmu-miR-1901_st | miR-1901 (and other miRNAs w/seed CGCUCGU) | -2.3333 | 0.0325668 | RF down vs. B6 |
| mmu-miR-193-star_st | miR-193a-5p (and other miRNAs w/seed GGGUCUU) | -3.01488 | 0.00314498 | RF down vs. B6 |
| mmu-miR-1938_st | miR-1938 (and other miRNAs w/seed GGUGGGA) | 2.30234 | 0.00456125 | RF up vs. B6 |
| mmu-miR-1945_st | miR-1945 (and other miRNAs w/seed CUUCGCG) | -2.22204 | 0.00292078 | RF down vs. B6 |
| mmu-miR-195_st | miR-16-5p (and other miRNAs w/seed AGCAGCA) | 2.40774 | 0.00013245 | RF up vs. B6 |
| mmu-miR-1958_st | miR-1958 (and other miRNAs w/seed AGGAAAG) | 2.33578 | 0.0112751 | RF up vs. B6 |
| mmu-miR-1963_st | miR-1963 (and other miRNAs w/seed GGGACGA) | 2.48985 | 0.00696223 | RF up vs. B6 |
| mmu-miR-200a-star_st | miR-200a-5p (and other miRNAs w/seed AUCUUAC) | -2.29475 | 0.0101925 | RF down vs. B6 |
| mmu-miR-2141_st | N/A | 3.24508 | 0.00279661 | RF up vs. B6 |
| mmu-miR-31_st | miR-31-5p (and other miRNAs w/seed GGCAAGA) | -2.00887 | 0.00273967 | RF down vs. B6 |
| mmu-miR-339-5p_st | miR-339-5p (and other miRNAs w/seed CCCUGUC) | -2.35516 | 0.0450179 | RF down vs. B6 |
| mmu-miR-3474_st | miR-3474 (and other miRNAs w/seed CCUGGGA) | 2.17607 | 0.0047377 | RF up vs. B6 |
| mmu-miR-423-3p_st | miR-423-3p (and other miRNAs w/seed GCUCGGU) | -2.21431 | 0.0292464 | RF down vs. B6 |
| mmu-miR-433-star_st | miR-433-5p (and other miRNAs w/seed ACGGUGA) | 2.37908 | 0.0375874 | RF up vs. B6 |
| mmu-miR-466b-3p_st | N/A | 2.49193 | 0.0291 | RF up vs. B6 |
| mmu-miR-466a-3p_st | miR-297a-3p (and other miRNAs w/seed AUACAUA) | 2.50884 | 0.0405124 | RF up vs. B6 |
| mmu-miR-466c-3p_st | N/A | 2.3573 | 0.0191953 | RF up vs. B6 |
| mmu-miR-494_st | miR-494-3p (and other miRNAs w/seed GAAACAU) | 2.79224 | 0.00087401 | RF up vs. B6 |
| mmu-miR-532-3p_st | miR-532-3p (and other miRNAs w/seed CUCCCAC) | -2.63149 | 0.00620057 | RF down vs. B6 |
| mmu-miR-540-3p_st | miR-540-3p (and other miRNAs w/seed GGUCAGA) | 2.39441 | 0.00586761 | RF up vs. B6 |
| mmu-miR-675-5p_st | miR-675-5p (and other miRNAs w/seed GGUGCGG) | 2.74952 | 0.0232645 | RF up vs. B6 |
| mmu-miR-698_st | miR-698-3p (and other miRNAs w/seed AUUCUCG) | -2.966 | 0.045387 | RF down vs. B6 |
| mmu-miR-712_st | miR-712-5p (and other miRNAs w/seed UCCUUCA) | -2.54359 | 0.0173272 | RF down vs. B6 |
| mmu-miR-761_st | miR-214-3p (and other miRNAs w/seed CAGCAGG) | 2.05608 | 0.0203094 | RF up vs. B6 |
| mmu-miR-877-star_st | miR-3909 (and other miRNAs w/seed GUCCUCU) | -2.15893 | 0.0177436 | RF down vs. B6 |
| mmu-miR-881-star_st | miR-881-5p (and other miRNAs w/seed AGAGAGA) | 2.43458 | 0.032487 | RF up vs. B6 |
| mmu-miR-210_st | miR-210-3p (and other miRNAs w/seed UGUGCGU) | -2.26924 | 0.00087604 | RF down vs. B6 |
| mmu-miR-466e-3p_st | miR-297a-3p (and other miRNAs w/seed AUACAUA) | 0.0211643 | 2.8393 | RF up vs. B6 |
| mmu-miR-34b-5p_st | miR-34a-5p (and other miRNAs w/seed GGCAGUG) | 0.0152598 | 2.15951 | RF up vs. B6 |
| mmu-miR-465a-3p_st | miR-465b-3p (and other miRNAs w/seed AUCAGGG) | 0.0249074 | 2.1084 | RF up vs. B6 |
| mmu-miR-465a-5p_st | miR-465b-5p (and other miRNAs w/seed AUUUAGA) | 0.0160314 | 3.40403 | RF up vs. B6 |
| mmu-miR-465b-3p_st | N/A | 0.0200618 | 2.24413 | RF up vs. B6 |
| mmu-miR-465c-3p_st | N/A | 0.024667 | 2.04989 | RF up vs. B6 |
| mmu-miR-465c-5p_st | N/A | 0.00554413 | 3.12558 | RF up vs. B6 |
| mmu-miR-467c_st | miR-467c-5p (and other miRNAs w/seed AAGUGCG) | 0.0264467 | 2.17301 | RF up vs. B6 |
| mmu-miR-467e_st | miR-467e-5p (and other miRNAs w/seed UAAGUGU) | 0.0321724 | 2.62692 | RF up vs. B6 |
| mmu-miR-493_st | miR-493-3p (and other miRNAs w/seed GAAGGUC) | 0.002434 | -3.13038 | RF down vs. B6 |
| mmu-miR-671-3p_st | miR-671-3p (and other miRNAs w/seed CCGGUUC) | 0.00427146 | -2.59383 | RF down vs. B6 |
| mmu-miR-741_st | miR-741-3p (and other miRNAs w/seed GAGAGAU) | 0.00774897 | 3.18463 | RF up vs. B6 |
| mmu-miR-743b-3p_st | miR-743b-3p (and other miRNAs w/seed AAAGACA) | 0.00570292 | 3.07961 | RF up vs. B6 |
| mmu-miR-883a-3p_st | miR-883-3p (and other miRNAs w/seed AACUGCA) | 0.0253477 | 2.38161 | RF up vs. B6 |
